# Retraction: miR-26a and its Target CKS2 Modulate Cell Growth and Tumorigenesis of Papillary Thyroid Carcinoma

**DOI:** 10.1371/journal.pone.0250313

**Published:** 2021-04-13

**Authors:** The PLOS ONE Editors

Following the publication of this article [1], concerns were raised regarding results presented in Figs 2, 3, 4 and 5. Specifically,

- Similarities have been detected within and between the following FACS panels presenting different experimental conditions:
  ○ The Fig 2G miR-26a-TCP-1 cell panel, the Fig 2G EV-TPC-1 cell panel, the Fig 4D miR-26a mimic panel, and the Fig 4D CKS2 siRNA panel.
  ○ The Fig 4D miR-26a control panel and the Fig 4D CKS2 siRNA control panel.
  ○ The Fig 5D CKS2 panel and the Fig 5D anti-miR-26a panel.
  ○ The Fig 5D EV panel and the Fig 5D anti-miR control panel.

Although these panels are not identical, they appear to be more similar than would be expected from independent samples.

- A vertical irregularity suggestive of gel splicing has been detected between the second and the third lane of the GAPDH panel of Fig 3C.

The underlying data to support the published results are no longer available.

In light of the concerns affecting multiple figure panels that question the integrity of these data, the *PLOS ONE* Editors retract this article.

XZ agreed with the retraction and apologizes for the issues with the published article. MiL, MaL, QC, MY, WL, LD, HC, DF, and ZL either did not respond or could not be reached directly.
